# Assessing the Presence of *Wuchereria bancrofti* Infections in Vectors Using Xenomonitoring in Lymphatic Filariasis Endemic Districts in Ghana

**DOI:** 10.3390/tropicalmed4010049

**Published:** 2019-03-17

**Authors:** Sellase Pi-Bansa, Joseph H. N. Osei, Worlasi D. Kartey-Attipoe, Elizabeth Elhassan, David Agyemang, Sampson Otoo, Samuel K. Dadzie, Maxwell A. Appawu, Michael D. Wilson, Benjamin G. Koudou, Dziedzom K. de Souza, Jürg Utzinger, Daniel A. Boakye

**Affiliations:** 1 Swiss Tropical and Public Health Institute, CH-4002 Basel, Switzerland; juerg.utzinger@swisstph.ch; 2 University of Basel, CH-4003 Basel, Switzerland; 3 Noguchi Memorial Institute for Medical Research, College of Health Sciences, University of Ghana, LG 581 Legon, Ghana; josei@noguchi.ug.edu.gh (J.H.N.O.); debikart@gmail.com (W.D.K.-A.); sotoo@noguchi.ug.edu.gh (S.O.); sdadzie@noguchi.ug.edu.gh (S.K.D.); mappawu@noguchi.ug.edu.gh (M.A.A.); mwilson@noguchi.ug.edu.gh (M.D.W.); ddesouza@noguchi.ug.edu.gh (D.K.d.S.); dboakye@noguchi.ug.edu.gh (D.A.B.); 4 Department of Animal Biology and Conservation Science, University of Ghana, LG 67 Legon, Ghana; 5 SightSavers International, Ghana Office, Accra, Ghana; lizzy.elhassan2@gmail.com (E.E.); dagyemang@sightsavers.org (D.A.); 6 Liverpool School of Tropical Medicine, Liverpool L3 5QA, UK; benjamin.koudou@lstmed.ac.uk; 7 Centre Suisse de Recherches Scientifiques en Côte d’Ivoire, 01 BP 1303, Abidjan 01, Côte d’Ivoire

**Keywords:** *Anopheles melas*, Ghana, lymphatic filariasis, post-mass drug administration surveillance, *Wuchereria bancrofti*, xenomonitoring

## Abstract

Mass drug administration (MDA) is the current mainstay to interrupt the transmission of lymphatic filariasis. To monitor whether MDA is effective and transmission of lymphatic filariasis indeed has been interrupted, rigorous surveillance is required. Assessment of transmission by programme managers is usually done via serology. New research suggests that xenomonitoring holds promise for determining the success of lymphatic filariasis interventions. The objective of this study was to assess *Wuchereria bancrofti* infection in mosquitoes as a post-MDA surveillance tool using xenomonitoring. The study was carried out in four districts of Ghana; Ahanta West, Mpohor, Kassena Nankana West and Bongo. A suite of mosquito sampling methods was employed, including human landing collections, pyrethrum spray catches and window exit traps. Infection of *W. bancrofti* in mosquitoes was determined using dissection, conventional and real-time polymerase chain reaction and loop mediated isothermal amplification assays. *Aedes*, *Anopheles coustani*, *An. gambiae*, *An. pharoensis*, *Culex* and *Mansonia* mosquitoes were sampled in each of the four study districts. The dissected mosquitoes were positive for filarial infection using molecular assays. Dissected *An. melas* mosquitoes from Ahanta West district were the only species found positive for filarial parasites. We conclude that whilst samples extracted with Trizol reagent did not show any positives, molecular methods should still be considered for monitoring and surveillance of lymphatic filariasis transmission.

## 1. Introduction

Lymphatic filariasis is a disease that occurs in tropical and subtropical parts of the world. The aim of the Global Programme to Eliminate Lymphatic Filariasis (GPELF), launched by the World Health Organization (WHO) in 2000, is to interrupt the transmission of lymphatic filariasis caused by *Wuchereria bancrofti* and *Brugia* species, and to manage morbidity and disability in affected individuals [1,2]. By 2011, guidelines had been developed and mass drug administration (MDA) was scaled up in 53 of the 73 lymphatic filariasis endemic countries [3], including Ghana. The Ghana Filariasis Elimination Programme (GFEP) was established in 2000 [4]. The inception was governed by preliminary data, indicating that lymphatic filariasis was endemic in 49 out of 110 districts. Microfilariae (mf) and immunochromatographic test (ICT) prevalence ranged between 19.8% and 29.6% and between 33.1% and 45.4%, respectively [4]. This led to the commencement of MDA in 2001 in 10 districts and the subsequent scaling up to the remaining endemic districts by 2006 [4,5]. Monitoring and evaluation (M&E) of the impact of MDA usually does not involve the detection of filarial larvae in mosquito vectors. Hence, xenomonitoring has not been officially part of WHO recommendations for lymphatic filariasis surveillance.

WHO put forth rigorous procedures for documenting interruption of lymphatic filariasis transmission in endemic countries [1]. These include mapping for the identification of endemic regions, followed by at least five rounds of annual MDA with periodic M&E. A transmission assessment survey (TAS) is conducted after the cessation of MDA and a 5-year post-validation to confirm that no recrudescence of lymphatic filariasis occurred [6]. Measuring progress of any lymphatic filariasis control programme is, however, dependent on the effectiveness of M&E post-MDA [7,8], among other issues. Monitoring of lymphatic filariasis transmission by programme managers mainly involves mf assays and antigen tests in the human populations. A challenge with this monitoring approach is the reluctance of individuals to provide samples [9] and its inability to provide a “real-time” estimate of the disease [3,7]. Xenomonitoring, which detects infection in vectors, could serve as a complementary diagnostic tool to serology. Xenomonitoring is convenient, non-invasive [7,9] and can be used to assess the progress of lymphatic filariasis control activities [3,10,11]. Dorkenoo and colleagues, in a study in Togo, demonstrated the possibility of using molecular xenomonitoring for post-lymphatic filariasis validation surveillance [6]. In their study, the feasibility of using large-scale xenomonitoring was demonstrated. Furthermore, the absence of *W. bancrofti* infections in *Anopheles gambiae* was observed during a post-validation molecular xenomonitoring survey in Togo. In the southern part of Ghana, a recent study revealed 0.9% *W. bancrofti* infection and 0.5% infectivity rates in *An. gambiae* following several rounds of MDA in endemic districts [12].

The purpose of the current study was to evaluate lymphatic filariasis transmission in vectors using dissection and molecular xenomonitoring as diagnostic tools. The study was implemented in four districts; two districts in northern Ghana and two districts in southern Ghana. The results complement existing information on *W. bancrofti* infections in vector mosquitoes, and provide additional evidence of the feasibility of using xenomonitoring for M&E and surveillance activities post-MDA.

## 2. Materials and Methods

### 2.1. Study Sites

The study was conducted in eight communities, selected from four districts in the Western and Upper East regions of Ghana. Two communities were selected from each district. In the Upper East region, Badunu and Navio Central were selected from Kassena Nankana West district, and Atampiisi Bongo and Balungu Nabiisi from the Bongo district. In the Western region, Antseambua and Asemkow were selected from Ahanta West district, while Ampeasem and Obrayebona were selected from Mpohor district. A map showing the study districts has been published elsewhere [13]. These sites were selected based on lymphatic filariasis prevalence data stemming from monitoring activities by the Ghana National Neglected Tropical Disease Programme unit of the Ghana Health Service (Table 1).

### 2.2. Mosquito Collection and Identification

Mosquito sampling spanning both the dry and rainy seasons was done for 13 months (from July 2015 to July 2016) in the four study districts. A detailed explanation of the three mosquito sampling methods (i.e., human landing collections, pyrethrum spray catches and window exit traps) used by community vector collectors (CVCs) has been described by Pi-Bansa et al. [14]. Mosquitoes sampled were morphologically and molecularly identified. In short, morphological identification of mosquitoes involved the observation of mosquitoes under a microscope and separation into various genera [15,16]. Deoxyribonucleic acid (DNA) extracted from the legs of *An. gambiae* was used for the identification of sibling species [17] and molecular forms within the *An. gambiae* complex [18,19].

### 2.3. Mosquito Dissection

The sample size of *An. gambiae* mosquitoes for dissection was specifically calculated for the various districts, as described by Naing et al. [20]. Mosquitoes were placed on a glass slide. A pair of dissecting pins was used to separate the head, thorax and abdomen. Then, a drop of normal saline was added to each segment. Dissection of mosquitoes and identification of the *W. bancrofti* larval stages was done under a microscope [21].

### 2.4. Extraction and Detection of *W. bancrofti* in Dissected Mosquitoes

All *W. bancrofti* negative and positive mosquitoes were scraped into Eppendorf tubes, pending further molecular analyses. The various mosquito species were grouped into pools ranging from 1–25. DNA was extracted from pooled mosquitoes using the Qiagen DNeasy tissue kit (Qiagen CA) extraction method, adhering to the manufacturer’s instructions. Extraction was followed by identification of parasite DNA in pooled mosquitoes using a loop-mediated isothermal amplification (LAMP) assay [11,22], conventional polymerase chain reaction (PCR) [23] and real-time (RT)-PCR [24]. These assays were performed using standard protocols described elsewhere [11,22,23,24]. Positive and negative controls were included in all reactions.

### 2.5. Extraction of Nucleic Acids from Pooled Mosquitoes with a TRIzol Reagent

Mosquitoes were randomly selected for the extraction of DNA and RNA using a TRIzol reagent (Life Technologies; Carlsbad, CA, USA). In order to estimate an infection rate of 1% with a power of 0.80, the estimated total number of mosquitoes required for each district was 2000 [6,25]. The protocol for determining infectivity required that samples were stored in RNAlater so as to enable RNA extraction from mosquitoes. *An. gambiae*, *Mansonia* and *Culex* species sampled by human landing catches and stored in RNAlater reagent (Life Technologies; Carlsbad, CA, USA) were pooled (range: 5–20). The determination of the number of mosquitoes in a pool was based on prior research pursued by Boakye et al., which tested different mosquito pool sizes (i.e., 25, 50, 100 and 200) [26]. Several additional studies had pools of mosquitoes of up to 30 specimens [9,11,27]. Extraction of DNA and RNA on pooled mosquitoes was done to assess both *W. bancrofti* infection and infectivity rates, respectively [27]. Detection of both infection and infectivity in pooled mosquitoes followed the protocols of Rao et al. [24] and Laney et al. [27]. Furthermore, quality control was done for the detection of infection in *An. gambiae* complex by extracting DNA from pooled Kisumu mosquitoes (laboratory reared susceptible *An. gambiae* strains, *n* = 20) spiked with 5–20 µL of *W. bancrofti* mf positive blood samples (57 mf/mL), which showed amplification for the parasite. The extraction protocol was replicated for this study (see Supplementary File).

### 2.6. Statistical Analysis

Data were entered into Microsoft Excel (Microsoft Corporation; Redmond, WA, USA). The Poolscreen 2.0 software (University of Alabama; Birmingham, USA) was used to calculate the maximum likelihood estimate of infection in the vector populations, along with the 95% confidence interval (CI) [28]. The various entomological indices assessed included vector biting density, infection and infectivity rates, annual/monthly transmission potentials and worm load in mosquitoes [29,30].

### 2.7. Ethical Approval

This study was approved by the institutional review board of the Noguchi Memorial Institute for Medical Research (Accra, Ghana; reference no. CPN 077/13-14, 7 May 2014) and the institutional research commission of the Swiss Tropical and Public Health Institute (Basel, Switzerland; reference no. FK 122a, 24 November 2015). All CVCs consented orally to participate in the study. Albendazole and ivermectin were administered to CVCs before mosquito sampling commenced. Arrangement was also made with the nurses at the community-based health planning and services compound to provide treatment for CVCs who reported at their facility and tested positive for malaria.

## 3. Results

### 3.1. Mosquito Abundance and Composition

A total of 31,064 mosquitoes were collected during the 13-month study period: 27,739 (89.3%) by human landing catches, 2687 (8.7%) by pyrethrum spray collections and 638 (2.1%) by window exit traps. The numbers of mosquitoes sampled from all districts using the various sampling techniques are summarised in Table 2. *An. gambiae* sensu lato (s.l.) (*n* = 23,102; 83.3%), the main lymphatic filariasis vector in Ghana, had the highest number collected using human landing catches. Other species collected included *Mansonia* spp. (*n* = 2474; 8.9%), *Culex* spp. (*n* = 2056; 7.4%), *Aedes* spp. (*n* = 92; 0.3%), *An. coustani* (*n* = 11; 0.04%) and *An. pharoensis* (*n* = 4; 0.01%). For pyrethrum spray collections, 1884 (70.1%) *An. gambiae*, 720 (26.8%) *Culex* spp., 40 (1.5%) *An. pharoensis*, 26 (1.0%) *Mansonia* spp., 10 *An. coustani* and 7 *Aedes* spp. were collected. A total of 562, 10, 3 and 1 mosquitoes were reported for *An. gambiae*, *An. pharoensis*, *Aedes* spp. and *An. coustani*, respectively, using window exit traps. *Culex* and *Mansonia* spp. had the same number (*n* = 31) sampled for window exit traps.

### 3.2. Molecular Identification of *An. gambiae* and *W. bancrofti*

A total of 320, 368, 217 and 211 *An. gambiae* s.l. from Ahanta West, Mpohor, Kassena Nankana West and Bongo districts, respectively, were identified at the molecular level. Results shown in Table 3 indicate high numbers of the sibling species *An. melas* in Ahanta West district. Relatively high numbers of *An. coluzzii*, formerly known as M form of the *An. gambiae* complex, were obtained from Mpohor, Kassena Nankana West and Bongo districts. Eight mosquitoes observed to be infected with *W. bancrofti* by dissection tested positive when pool screened using PCR.

### 3.3. Transmission Indices of *An. gambiae* Complex from Ahanta West District

**Figure 1:**
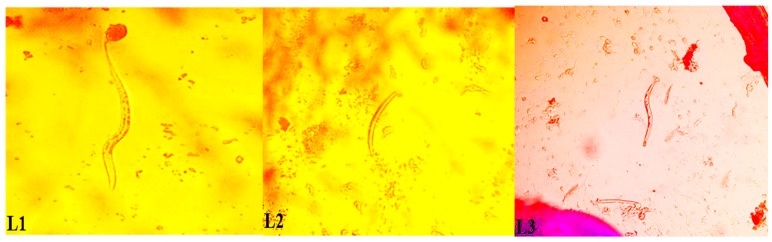
Three larval stages of *W. bancrofti* parasites from Ahanta West district in Ghana.

The average vector biting density for *An. gambiae*, sampled using human landing collections from Ahanta West, Mpohor, Kassena Nankana West and Bongo districts, were 43.8, 9.9, 1.0 and 0.8 bites/person/night, respectively. *W. bancrofti* infections were reported only in *An. melas*, a sibling species within the *An. gambiae* complex from Ahanta West district for this study. Eight *An. melas* mosquitoes were found infected (harbouring any of the developmental stage(s) of the parasite: mf, larval stages 1 (L_1_), 2 (L_2_) or 3 (L_3_), of which two mosquitoes were infective, harbouring only L_3_, as shown in Figure 1. The total numbers of L_1_, L_2_ and L_3_ counted from all the slides were 10, 2 and 2, respectively. The monthly infective biting rates (MIBR) and the annual infective biting rate (AIBR) were 8.0 and 95.9 infective bites/person, respectively. The annual transmission potential (ATP) due to *An. gambiae* in the Ahanta West district was 7.4 (Table 4).

### 3.4. Detection of *W. bancrofti* Using Molecular Techniques

A total of 2000 *An. gambiae* s.l. from Ahanta West and Mpohor districts, 253 from Kassena Nankana West and 225 from Bongo districts were screened for *W. bancrofti* infections and infectivity using RT-PCR. None of the 4478 *An. gambiae* processed in 214 pools from all study districts were found positive for *W. bancrofti*. Screening was also done for both *Mansonia* and *Culex* species from the four districts, though very few numbers were sampled from Mpohor, Kassena Nankana West and Bongo, compared to Ahanta West. Both *Mansonia* and *Culex* species were found negative for *W. bancrofti* in all districts (Table 5). All dissected mosquitoes from the four districts that were negative for *W. bancrofti* parasite and further screened by LAMP, conventional PCR and RT-PCR tested negative.

## 4. Discussion

Rigorous monitoring of *W. bancrofti* infections in mosquito vectors after several rounds of MDA is recommended to provide information on the progress of control and elimination activities. Indeed, such monitoring activities are necessary for making programmatic decisions that will eventually lead to certification of lymphatic filariasis elimination in previously endemic regions [26]. The current study, which was part of an operational research project to determine reasons for persistent lymphatic filariasis transmission in selected districts of Ghana after more than 10 rounds of MDA, investigated the feasibility and usefulness of a xenomonitoring approach for post-MDA surveillance to assess filarial infections in vectors [22,31]. Our study also provides information on the lymphatic filariasis infection status in vectors after multiple rounds of MDA in previously endemic districts.

The sampling methods used for this study included human landing collections, pyrethrum spray catches and window exit traps. These collection methods have been used before for sampling mosquitoes for xenomonitoring activities [14,32,33,34]. Recently, the Ifakara tent trap has been reported as an alternative to human landing collections and it was emphasised that it exhibits an improved ethical profile [35,36]. However, at the time our study was implemented, we did not have access to the Ifakara tent trap. Results from our study revealed high numbers of *An. gambiae* complex, the primary lymphatic filariasis vector in Ghana [9,37], in all four districts. The highest density was observed in Ahanta West district. The high densities of vectors and observed infections (L_1_, L_2_ and L_3_) in Ahanta West district might explain the presence of *W. bancrofti* infection in the *An. gambiae* complex from this district. A relatively higher density of *An. gambiae* was recorded in Kassena Nankana West district, compared to Bongo district. Both districts are in the dry Guinea savannah ecological zone [29], whilst the Ahanta West and Mpohor districts are situated in the rain forest ecological zone [38]. In the year 2000, high baseline mf prevalences of 19.5% and 29.4% were reported in Ahanta West and Kassena Nankana West districts, while considerably lower mf prevalences were observed in 2014; 2.7% and 1.3%, respectively (Table 1), after multiple rounds of MDA. The present study recorded *W. bancrofti* infection rates of 0.025 and nil for Ahanta West and Kassena Nankana West districts, respectively (Table 4). These very low infection rates observed in mosquitoes from this study correspondingly reflect the low lymphatic filariasis prevalence rates in the human population. Moreover, the availability of efficient vectors (*An. melas* and/or *Mansonia* spp.) in all four study districts can lead to picking up *W. bancrofti* infections, even at low parasitaemia, as seen in the Ahanta West district. Despite the large numbers of efficient vectors in a given district, the very low rates or the absence of *W. bancrofti* infections in the human population is likely to result in the absence of infections in vectors. Hence, there should be enough *W. bancrofti* parasites in the blood of human population for vectors to successfully ingest after a blood meal, since at very low mf levels, vectors are unlikely to ingest parasites. This may explain the absence of infections in the large number of *An. gambiae* vectors collected and examined in Kassena Nankana West, Mpohor and Bongo districts.

Furthermore, results from molecular species identification of the *An. gambiae* complex showed a high proportion of *An. coluzzii* (formally the M form of *An. gambiae* complex) in almost all districts (Table 3). This could be associated with the fact that *An. coluzzii*, which prefer breeding in ephemeral sites like run-off and flood water, are mostly found in the northern and coastal savannah areas of Ghana where this study was conducted [39]. Kassena Nankana West district recording the highest number of *An. arabiensis* could possibly be due to its location in the northern part of Ghana where the climate is arid, which represents the preferred breeding condition for this mosquito species [19]. *An. melas*, which is a sibling species within the *An. gambiae* complex, was mostly found in the Ahanta West district, corroborating previous findings by Dunyo et al. [40]. *Anopheles* mosquitoes are known to exhibit “facilitation”, which makes it possible for these mosquito species to pick up *W. bancrofti* parasites at high mf rates in the human population and develop them to the infective stage [41,42,43]. However, *An. melas* exhibits “limitation”, and hence this species can ingest and develop mf to the infective stage, even at low parasite densities [42,43]. In view of the high numbers of *An. melas* recorded in Ahanta West district, it is conceivable that this species is responsible for the observed *W. bancrofti* parasites (L_1_, L_2_ and L_3_).

The ABR for *An. gambiae* complex was highest in the Ahanta West district (15,987 bites/person/year). Finding *W. bancrofti* infections in Ahanta West district may be due to the high number of lymphatic filariasis vectors, specifically from the *An. gambiae* complex with a reported prevalence of 2.7% in this district. In the Kassena Nankana West district, before the commencement of our study, the reported prevalence of 1.3% was indicative of low persistent lymphatic filariasis transmission. A possible reason for the absence of infections in Kassena Nankana West is the relatively low level of infection in the human population. Another factor is the lower ABR (376.3 bites/person/year) in this district. There were no *W. bancrofti* infections recorded in the Mpohor and Bongo districts. This may have been due to the zero mf prevalence reported for these two districts (Table 1) before the onset of this study. In a previous study, Appawu et al. [29] investigated the entomological role played by the two lymphatic filariasis vectors *An. gambiae* and *An. funestus* at irrigation project sites in the Upper East region of Ghana. The authors recorded *W. bancrofti* infections in all study districts. Their results indicated that for irrigated communities like Tono and Vea, higher vector densities resulted in more infective feeds compared to Azoka, a non-irrigated community. The 7.4 ATP of *An. gambiae* in the Ahanta West district was due to the observed L_3_ in *An. melas* and reported mf-positive individuals from this district. The ATP value of *An. gambiae* obtained in spite of the low infectivity rate might be explained by the large number of *An. gambiae* collected in this district. In our study, the vector observed having *W. bancrofti* infections (L_1_, L_2_ and L_3_) was only *An. melas* belonging to the *An. gambiae* complex. Additionally, identification of *Mansonia* species in Ahanta West district suggests that these vectors could take up mf and successfully develop them to the infective stage, even at low parasitaemia [12,44].

Mosquito vector control activities reduce vector densities and human-vector contact [45,46]. This in turn decreases the likelihood of vectors picking up *W. bancrofti* parasites in endemic areas that have undergone several rounds of MDA. We found considerably higher bednet usage in Mpohor and Bongo districts, compared to Ahanta West and Kassena Nankana West districts. Hence, there was higher human–vector contact in the latter two districts. This could have contributed to the high ABR recorded for Ahanta West leading to *W. bancrofti* infections in the vectors due to infections in the human population. Though Mpohor district had recorded a relatively high ABR (3604), the reported prevalence of zero may explain the absence of infections in this district.

Additionally, mosquito species previously considered as non-vectors might be acting as vectors of lymphatic filariasis as in the case of *Mansonia* in Ghana [47] and *Culex* in Nigeria [48,49]. This observation, together with the fact that parasite DNA can be detected in both vector and non-vector mosquitoes [6], led to the investigation of both species in this study. No positive result was recorded for culicines using molecular assays. Furthermore, molecular assays run on DNA and RNA extracted from selected *An. gambiae* complex from the various districts was negative for filarial infections. The absence of infections in *An. gambiae* complex could have been as a result of PCR inhibition due to the masking of parasite DNA by mosquito DNA due to extraction of pooled mosquito samples.

## 5. Conclusions

Our study, employing xenomonitoring as a post-MDA surveillance tool, revealed that, at low parasitaemia, infections are usually found and sustained in vectors that exhibit limitation as seen in Ahanta West district. Additionally, *An. melas* emerges as an important vector for xenomonitoring along the coastal communities of the Western region in the southern part of Ghana. Moreover, effective vector control activities like high coverage of bednets can decrease ABR values in any endemic foci. As revealed in our previous work, vector control activities (bednet usage) in Mpohor and Bongo districts were relatively high. The reported zero prevalence of human infections and reduction in the human vector contact due to bednet usage might be responsible for the absence of infections in mosquito vectors from these districts. Presently in Ghana, only little emphasis is placed on the inclusion of xenomonitoring in decision-making processes during lymphatic filariasis programmatic activities. As shown here, data from xenomonitoring could be used by programme managers and other stakeholders to support decisions of stopping or continuing MDA. Additionally, complementing vector control activities with MDA during lymphatic filariasis control activities could reduce *W. bancrofti* infections in mosquitoes.

## Figures and Tables

**Table 1:** Number of mass drug administration (MDA) rounds and prevalence of microfilariae in the four districts of Ghana where the current study was conducted between July 2015 and July 2016.

District	Community	Number of MDA Rounds	Microfilariae Prevalence in 2000 (%)	Microfilariae Prevalence in 2014 (%)	Number of *An. gambiae* Dissected
**Ahanta West**	Asemkow Antseambua	16	19.5	2.7	320
**Mpohor**	Obrayebona Ampeasem	11	0.0	0.0	368
**Kassena Nankana West**	Navio Central Badunu	15	29.4	1.3	217
**Bongo**	Atampiisi Bongo Balungu Nabiisi	13	16.7	0.0	211

**Table 2:** Mosquitoes sampled using three different sampling methods from four study districts in Ghana during a 13-month sampling period between July 2015 and July 2016.

Method	Community	District	*An. gambiae*	*Culex* species	*Mansonia* species	*Aedes* species	*An. pharoensis*	*An. coustani*	Total Collected
**Human landing catches**	Asemkow Antseambua	Ahanta West	18,213	1200	2386	8	0	4	
	Obrayebona Ampeasem	Mpohor	4109	66	72	6	0	3	
	Badunu Navio Central	Kassena Nankana West	426	489	11	42	2	4	
	Atampiisi Bongo Balungu Nabiisi	Bongo	354	301	5	36	2	0	
**Pyrethrum spray catches**	Asemkow Antseambua	Ahanta West	271	4	19	1	36	0	
	Obrayebona Ampeasem	Mpohor	375	14	7	0	1	0	
	Badunu Navio Central	Kassena Nankana West	801	384	0	1	1	9	
	Atampiisi Bongo Balungu Nabiisi	Bongo	437	318	0	5	2	1	
**Window exit trap**	Asemkow Antseambua	Ahanta West	396	17	29	0	0	0	
	Obrayebona Ampeasem	Mpohor	119	1	1	1	9	0	
	Badunu Navio Central	Kassena Nankana West	12	6	1	1	1	0	
	Atampiisi Bongo Balungu Nabiisi	Bongo	35	7	0	1	0	1	
**Total**			25,548	2807	2531	102	54	22	31,064

**Table 3:** Distribution of members of the *An. gambiae* complex in four study districts, Ghana, collected between July 2015 and July 2016.

District	Sibling Species of the *Anopheles gambiae* Complex							
	*An. gambiae* s. s.		*An. arabiensis*		*An. melas*		*An. coluzzii*	
	n	%	n	%	n	%	n	%
Ahanta West	3	0.9	11	3.4	275	85.9	12	3.8
Mpohor	226	61.4	0	0	1	0.3	122	33.2
Kassena Nankana West	57	26.3	25	11.5	0	0	124	57.1
Bongo	54	25.6	0	0	0	0	142	67.3

**Table 4:** Entomological indices showing relevant parameters for the estimation of the annual transmission potential (ATP).

District	Average Number of *An. gambiae* Sampled per Month	Vector Biting Density (MBR)	Annual Biting Rate (ABR)	Average Number of *An. gambiae* Dissected per Month	Average Infection per Month	Average Infectivity per Month	Infection Rate (%)	Infectivity Rate (%)	Annual Infective Biting Rate (AIBR)	Average Worm Load per Month	Annual Transmission Potential (ATP)
Ahanta West	1401	43.8	15,987	25	0.620	0.150	0.025 (2.5)	0.006 (0.6)	95.922	0.077	7.386
Mpohor	316	9.9	3614	28	0	0	0	0	0	0	0
Kassena Nankana West	33	1.0	365	17	0	0	0	0	0	0	0
Bongo	27	0.8	292	16	0	0	0	0	0	0	0

**Table 5:** Number of mosquito pools processed per study district from July 2015 to July 2016.

Species	District	Number of Pools	Average Pool Size	Number of Mosquitoes Processed	Positive (Infection/Infectivity)	95% CI
*An. gambiae*	Ahanta West	97	20.6	2000	0	0–0.00095
	Mpohor	91	22.0	2000	0	0–0.00095
	Kassena Nankana West	13	19.5	253	0	0–0.00756
	Bongo	13	17.3	225	0	0–0.00849
*Mansonia* species	Ahanta West	83	21.1	1754	0	0–0.00109
	Mpohor	2	25.0	50	0	0–0.03767
	Kassena Nankana West	1	14.0	14	0	0–0.12815
	Bongo	1	5.0	5	0	0–3.18868
*Culex* species	Ahanta West	63	20.0	1261	0	0–0.00152
	Mpohor	2	19.0	38	0	0–0.04927
	Kassena Nankana West	19	19.4	369	0	0–0.00518
	Bongo	8	16.3	133	0	0–0.01433

## Supplementary Materials

The following are available online at https://www.mdpi.com/2414-6366/4/1/49/s1, Figure S1. Amplification of *Wuchereria bancrofti* DNA from pooled laboratory reared susceptible Kisumu mosquitoes. The diagram shows amplification curves for four different pools (*n* = 20) of susceptible Kisumu mosquitoes and a positive control. The four pools were spiked with 5–20 µL of *Wuchereria bancrofti* microfilariae positive blood having a concentration of 57 mf/mL.
