# Supplementary figures and images for: Assessing the Presence of Wuchereria bancrofti Infections in Vectors Using Xenomonitoring in Lymphatic Filariasis Endemic Districts in Ghana
Source: Trop Med Infect Dis. 2019 Mar 17;4(1):49. doi: 10.3390/tropicalmed4010049 (PMC6473662; doi:10.3390/tropicalmed4010049)

Delta Rn vs Cycle

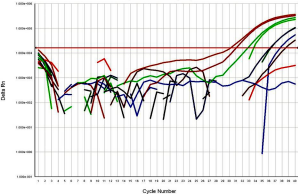

Supplement: Supplementary file 1 [file tropicalmed-04-00049-s001.pdf]
